# Do Young Consumers Care about Antioxidant Benefits and Resveratrol and Caffeic Acid Consumption?

**DOI:** 10.3390/nu16101439

**Published:** 2024-05-10

**Authors:** Cristina Ștefania Gălbău, Mihaela Badea, Laura Elena Gaman

**Affiliations:** 1 Department of Fundamental, Prophylactic and Clinical Disciplines, Faculty of Medicine, Transilvania University of Brasov, 500036 Brașov, Romania; cristina.adochite@unitbv.ro; 2 Research Center for Fundamental Research and Prevention Strategies in Medicine, Research and Development Institute of Transilvania University of Brasov, 500484 Brașov, Romania; 3 Faculty of Medicine, “Carol Davila” University of Medicine and Pharmacy, 020021 Bucharest, Romania; gaman.laura@umfcd.ro

**Keywords:** antioxidants, resveratrol, caffeic acid, survey

## Abstract

Resveratrol and caffeic acid are some of the most consumed antioxidants during the day, so their importance as sources and their benefits need to be evaluated and updated. This survey aimed not only to analyze whether young Romanian consumers are informed about the benefits of antioxidants in general, and resveratrol and caffeic acid in particular, but also to observe the degree of nutritional education of these participants. Young consumers know the concept of antioxidants relatively well; they managed to give examples of antioxidants and indicate their effects. The majority of those chosen drink wine and coffee, but many are unaware of their health advantages and antioxidant properties. Students are less familiar with the antioxidant chemicals resveratrol and caffeic acid. It is advised to have a thorough understanding of these significant antioxidants and their nutritional content as they are present in our regular diets, and further studies on different kinds of antioxidants are required to increase the awareness of people concerning their importance in daily life.

## 1. Introduction

### 1.1. Resveratrol

**Figure 1:**
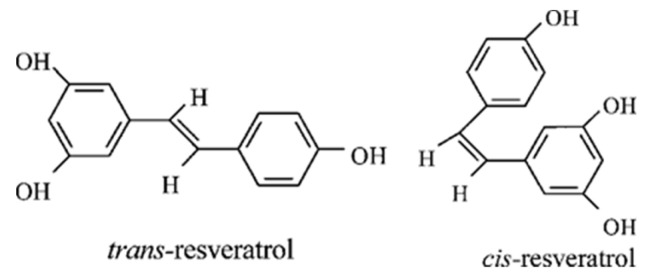
Chemical structure of trans-resveratrol and cis-resveratrol.

Resveratrol, the active compound found in red grapes and other plants, has a long history dating back to ancient Indian medicine. Grape extract was used for its health benefits as early as 2500 BC [1]. The peel of fresh grapes contains a significant amount of resveratrol (0.2–7.7 mg/L), and it is also found in wine. Resveratrol is now recognized as a polyphenol that can be found in various plants, not just grapes [2]. It has the potential to be transformed into active forms through metabolic processes (Figure 1), which enhances its therapeutic effects [3].

**Figure 2:**
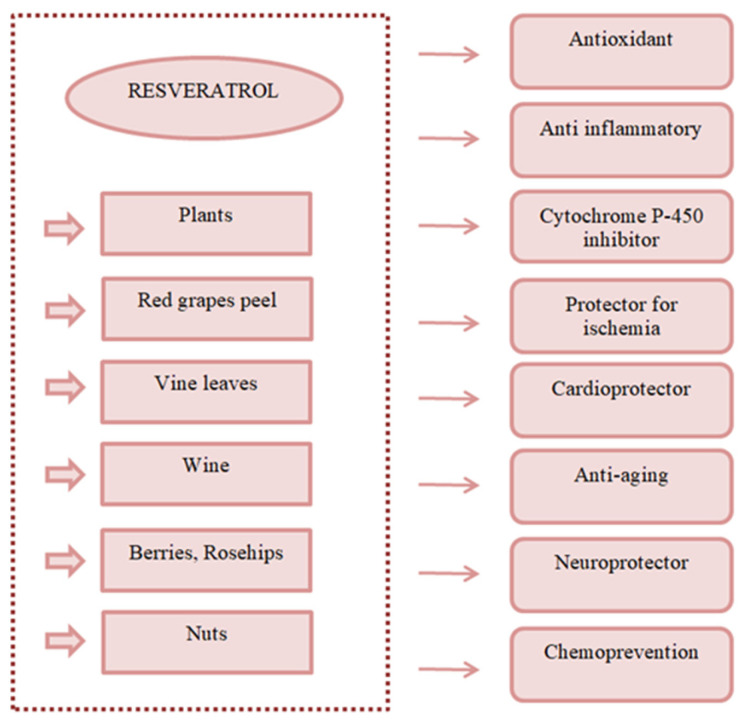
Sources of resveratrol and its health benefits.

Resveratrol has been identified in extracts from various plants and as synthetic derivatives. This compound has been identified in the skin of red grapes, vine leaves, blueberries, rosehips, hazelnuts, and wine [4]. Resveratrol is consumed in the form of natural food supplements and the form of herbs in Chinese medicine and Ayurvedic medicine in India [1]. In many studies, resveratrol has been indicated to have numerous biological functions: anti-inflammatory, antioxidant, anti-obesity, antibacterial [5,6]; it has also been reported for its anticancer activity for some forms of cancer, such as ovarian, breast, and prostate cancer [7] (Figure 2).

Its pharmacokinetic properties are not exactly the most favorable, because resveratrol is very slightly soluble in water. At the same time, it has been shown that its metabolites have a short half-life [8].

### 1.2. Caffeic Acid

**Figure 3:**
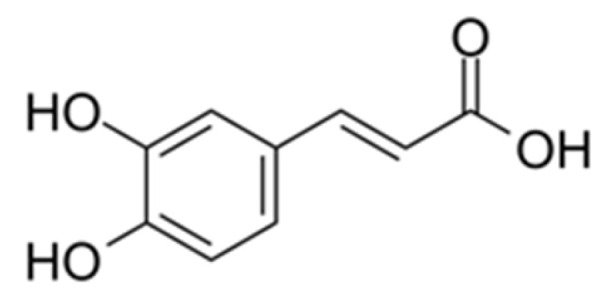
Chemical structure of caffeic acid.

Hydroxycinnamic acids are commonly found in plants and plant products. They can be created from phenylalanine and play a role in the production of other complex phenolic compounds like flavonoids [9,10]. Additionally, they contribute to the structure and support of plants by being part of the cell wall. In plants, these acids can exist as esters or be converted into other forms. The most prevalent hydroxycinnamic acids in nature are caffeic acid, its ester (chlorogenic acid), and its precursor p-cumaric acid [11]. Caffeic acid (Figure 3), which is a phenolic compound, is the main hydroxycinnamic acid in the human diet and can be found in coffee beans, teas, propolis, wines, and various fruits [12,13].

**Figure 4:**
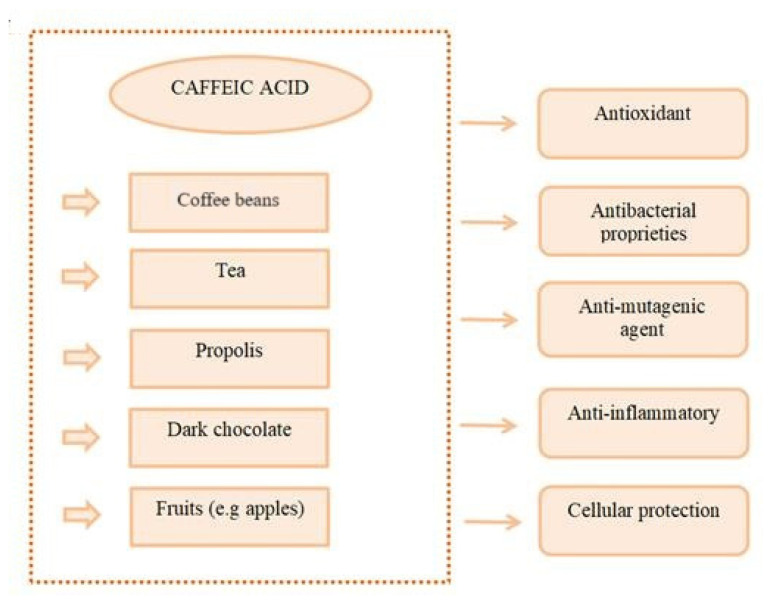
Sources of caffeic acid and its health benefits.

Caffeic acid is found in fruits, oil, vegetables, coffee, and dark chocolate [13,14]. It is a phenolic compound with pharmacological properties found mainly in medicinal plants (Figure 4). Caffeic acid has anti-inflammatory [15], anticancer [16], antibacterial [17], and antioxidant properties [18].

Regarding the toxic risk, it is considered non-existent because it is usually ingested in small amounts through plants. Most of the hydroxycinnamic acids ingested per day are caffeic acid. After ingestion, phenolic compounds are converted to methylated derivatives or can be conjugated to glucuronic acid (e.g., conversion of caffeic acid to ferulic acid) [19]. Free ferulic, caffeic, and coumaric acids have been identified in urine after a fruit-rich diet [20].

Drinking red wine or coffee, even without caffeine, exposes us to a natural polyphenolic component called caffeic acid, which has a wide range of intriguing health consequences. Those who drink a lot of coffee may take in up to 500 mg of caffeine daily; those who do not can take in up to 25 mg [21,22].

The main purpose of this questionnaire study is to analyze whether young consumers (students) are informed about the benefits of antioxidants in general, and resveratrol and caffeic acid in particular. The secondary purpose of this study is to observe the degree of nutritional education of young consumers.

## 2. Materials and Methods

### 2.1. Study Design

For this type of study considering young people, 135 students from three faculties (Faculty of Medicine—MD, Faculty of Food and Tourism—FT, and Faculty of Electrical Engineering and Computer Science—EECS) of the Transilvania University of Brașov were selected (50 students from MD and FT faculties and 35 students from EECS). MD and FT faculties aim, through the offered educational program, to improve public health and impart certain notions about antioxidants and their benefits. We included a third group of study to minimize the bias of the study, introducing a group that has minimal general knowledge from their previous educational program and probably medium knowledge about antioxidants in general. A special interest was to consider if the intake habits are different between these three groups of young Romanian consumers of antioxidants, as the base of discussion for other similar studies performed in other different cultural backgrounds, social statuses, and lifestyle habits.

This study complied with the rules of medical scientific ethics. Participants in this study were informed about the purpose of data processing, the anonymity of respondents, and the possibility to waive its completion without any additional problems. All students selected for this study completed at least 80% of the questionnaire.

### 2.2. Questionnaire Structure

The questions focused on topics from several medical fields (biochemistry, nutrition, internal medicine, and oncology) but also about the personal lifestyle of the respondents—to observe the degree of nutritional education and healthy or less healthy habits of students.

The response format was of two types: grid questions with at least five answer options and questions with a Likert scale (e.g., ‘strongly agree’, ‘agree’, ‘disagree’, ‘strongly disagree’). The answers to the questions were processed using the Office Excel 2016 package, and the results were statistically analyzed, compared, and reported as absolute numbers, and also as a percentile from the total number of their group of study, and presented in the form of suggestive tables that allow the comparison of batch answers.

## 3. Results

### 3.1. Group Characteristics

The young groups from Faculty of Medicine and Faculty of Food and Tourism had more women (76.0%—MD and 86.0%—FT), as is normal for each year in that type of faculty, and the Faculty of Electrical Engineering and Computer Science had 91.43% men. The average age for the MD group was 24.42 ± 7.18 years, for FT was 22 ± 1 years, and for EECS was 20.8 ± 0.9 years. Most of these young people were unmarried: 82.0% MD, 90% FT and 100% EECS.

### 3.2. General Knowledge about Antioxidants

A comparative study of the knowledge about antioxidants was conducted (Table 1).

The role of antioxidants in human disease pathogenesis is confirmed by an additional broad amount of the literature outside science book publishers. Phenolic compounds, especially flavonoids, can boost cell survival by acting as antioxidants; as pro-oxidants, they induce apoptosis and prevent cancer cell development [23].

### 3.3. Data concerning the Healthy Eating Behavior of the Respondents

Using a scale of 1–7, the young consumers were questioned concerning their healthy nutrition behavior [24] (Table 2).

This table shows the tendencies of volunteers towards a healthy or less healthy diet. In total, 40.0% of them expressed total disagreement over how unhealthy snacks are; 50.0% of them rated a score of 7 (total agreement) for ‘I’m interested in how healthy is what I eat’.

### 3.4. Knowledge concerning Resveratrol

The presence of a double bond in resveratrol allows for two different forms, trans and cis, which are referred to as diasteroisomers E and Z. Trans-resveratrol is more stable and preferred, especially in high pH and light conditions. On the other hand, cis-resveratrol is only stable in neutral pH and light-deprived conditions. Research has shown that the initial structure of resveratrol is crucial for its therapeutic effects. Substituting hydroxyl groups with methoxy enhances its cytotoxic activity, and having a hydroxyl group in the trans position at 4- and 4′- is important for its anti-proliferative effects. Introducing halogen groups could offer insights into its antibacterial activity [25].

The young participants were questioned concerning their knowledge about resveratrol, and the answers are indicated in Table 3.

### 3.5. Wine Consumption

The young students were questioned about the general consumption of alcoholic beverages, and particularly wine, and their answers are indicated in Table 4 and Table 5.

Some studies examine the factors that influence consumers’ wine preferences, including acidity, aroma, intensity, and how long the aroma lasts. An article discusses how the levels of chlorophyll in the leaves and the amount of water in the leaves before sunrise, measured during the grapes’ ripening stage, affect the wine’s chemical and sensory properties. The presence of iron stress lowers the pH of the wine and improves sensory qualities like tone, intensity, and flavor, with a greater impact than the water levels [26].

### 3.6. Knowledge about Caffeic Acid

The presence of phenolic groups and the substituents on these groups affect the antioxidant activity of these compounds. When the phenolic group undergoes oxidation, it forms a phenoxy radical which can then either be further oxidized to form quinone or combine with another phenoxy radical to form a stable dimer. Compounds with a catechol group have higher antioxidant capacity compared to those with only one phenolic group. Additionally, compounds with double bonds that extend the conjugation of the phenolic nucleus also have increased antioxidant capacity because they stabilize the formed radical. On the other hand, compounds with a saturated side chain or a hydrogen atom inhibit antioxidant activity [11].

In our study, some questions were addressed about the sources, benefits, and preferred forms of consumption of caffeic acid, and the obtained data are indicated in Table 6.

### 3.7. Coffee Consumption

Coffee has been highly valued for its natural and beneficial effects on the body and mind since ancient times. Trained experts have evaluated the qualities of coffee using questionnaires, but biases can occur. A recent article explored the use of wearable sensors to examine the emotional responses of experienced coffee judges. This technology-based approach found significant correlations between biological signals and survey data in the areas of taste, vision, and smell [27].

It was also interesting to know the reason and the amount of coffee consumption among our groups, and their answers are indicated in Table 7.

## 4. Discussions

Biological systems have developed many antioxidant pathways to monitor oxidative processes in addition to exogenous phenolic compounds. These processes involve antioxidant enzymes that are involved in the catalyzed elimination of superoxide and hydrogen peroxide, such as superoxide dismutase (SOD), catalase, and peroxidase. Glutathione transferase, ceruloplasmin, hemoxygenase, and probably several other enzymes can participate in the enzymatic monitoring of oxygen radicals and their properties, in addition to the three enzymes mentioned above. Non-enzymatic mechanisms also occur within biological systems, aside from enzymatic antioxidant systems. That includes E and C vitamins and glutathione. The primary lipid-soluble chain-breaking antioxidant in body tissues is vitamin E (tocopherols and tocotrienols), which plays a significant role in the first line of membrane defence against oxidative damage at the early stages of free radical assault. One of the most important intracellular barricades against degradation by reactive oxygen species is glutathione [28].

In addition to wine, fruit juices and milk, drinks contain a blend of many antioxidant compounds, such as vitamin C, carotenoids and phenolic compounds, as well as proteins. The most important contributions to antioxidants in the diet are by vegetables, mostly due to the excess of vitamins, phenolic compounds and carotenoids. Other tests also have shown that certain milk components (whey, casein, lactoferrin and albumin) have antioxidant activity [29]. The presence of effective oxygen radical scavengers such as vitamin C, carotenoids and phenolic compounds is correlated with the antioxidant properties of fruits and vegetables [28].

Antioxidants are specific compounds that can stop the oxidation of other substances and reduce the effects of free radicals. Antioxidants are compounds that can be synthesized in the human body, but we can mainly obtain them from ingested food [30]. It was observed that students from MD and FT faculties knew that antioxidants are specific compounds that have the role of reducing the effects of free radicals by 68%—FT and 66.0%—MD, but from EECS, only 20% chose this response. A bigger difference can be seen in the answer “Compounds we can take from food”, chosen by 48.0% of those from the Faculty of Medicine, 25.7% from Electrical Engineering and Computer Science, and only 10.0% of those from the Faculty of Food and Tourism (Table 1).

Examples of antioxidants indicated in the scientific literature are vitamin E, coenzyme Q, resveratrol, caffeic acid, glutathione peroxidase, vitamin C, SOD (superoxide dismutase), and vitamin A [31]. Following the analysis of Table 1, it appears that study groups chose in large percentages, 66% of the students of the Faculty of Medicine, 68.0% of the students of the Faculty of Food and Tourism, and 57.1% of the students from Electrical Engineering and Computer Science, that vitamin C is an antioxidant. For resveratrol, the results obtained indicate 44.0% of FT, 40.0% of EECS and 52.0% of MD students knew that resveratrol is an antioxidant. Also, for caffeic acid, the following results were obtained: half of the group of the Faculty of Food and Tourism, 48.0% of the students of the Faculty of Medicine and 60% of students from Faculty of Electrical Engineering and Computer Science knew that caffeic acid is an antioxidant (Table 1).

The literature indicates sources of antioxidants—eggs, chocolate, beans and carrots [31]. From all groups of young people, a small percentage chose hydrogenated oils, carbonated beverages and refined sugar as a response option for antioxidant sources. Most of the consumers indicated carrots as a source of antioxidants: 82.0%—MD and 86.0%—FT, and after that, a good percentage indicated beans, chocolate, and eggs as a source (Table 1).

The average overall resveratrol concentration in red wine is 7 mg/L, in rose wine is 2 mg/L and in white wine 0.5 mg/L, although it varies greatly according to grape variety, geographical indication and vintage [32]. The concentration of resveratrol in wine varies depending on the type of grape, location and vintage, with Merlot wines having the highest concentration and the local Prokupacac cultivar having the lowest. Specialized clinical studies have found that resveratrol has various benefits, including preventing aging, protecting the brain, reducing obesity, protecting the heart and reducing inflammation [33]. Most of these positive effects are seen in the cardiovascular system [32]. A dose of at least 30 mg/day of resveratrol has been found to stimulate endothelial activity in healthy individuals [32]. Different doses of resveratrol have also been correlated with improvements in other processes and parameters, as well as a decrease in pain score for women with dysmenorrhea (30 mg/day) and improvements in inflammatory biomarkers for ulcerative colitis (500 mg/day) [32].

According to the literature, the sources of resveratrol are grapes, hazelnuts, blueberries, cranberries and mulberries [31]. From the analysis of Table 3, it was observed that both groups know the main source of resveratrol, grapes—68.0% of the FT group, 42.86% of the EECS and 70.0% of the MD group. In total, 48.0% of MDs chose blueberries as a source of resveratrol, 25.71% of EECSs and those of FT, only 22.0%. A total of 30.0% of those from MD and only 20.0% of those from FT know cranberries as the primary source of resveratrol, but the respondents from EECS chose this response in a percentage of 45.71%.

Resveratrol has a short half-life of around 1.5 h due to its rapid absorption in the intestine and breakdown in the liver. When ingested, about 77–80% of resveratrol enters the bloodstream by passing through the cells lining the intestines. It then attaches to albumin and lipoproteins before easily being released and taken up by cells. Approximately 49–61% of resveratrol is excreted in the urine [31].

Table 3 shows the highest percentage for MD, namely, 54%, for the significant benefit of antioxidants and resveratrol, the prevention of ageing. To this, the EECS group chose to respond only in a percentage of 11.43%. At similar percentages, the groups chose that resveratrol is a good anti-inflammatory: 50% FT, 46.0% MD, 42.86% EECS.

Supported by clinical trials conducted across the types to accurately observe the therapeutic benefits of resveratrol, we selected a few of these, such as chemotherapeutic benefits in breast and lung cancer by inhibiting the growth of malignant tumors, such as those in the breast, colon, leukemia and myeloma, and antibacterial and anti-HIV properties [29] in idiopathic pulmonary fibrosis, asthma, chronic obstructive pulmonary disease and pneumonia [32]. Table 3 shows that 12% of FT did not answer this question at all, so it can be concluded that they have not heard or learned about resveratrol in terms of medical benefits. The percentage of responses from the group of students from MD is satisfactory; 50% knew that resveratrol inhibits the growth of malignant tumors and 45.71% from EECS. For short-term dosages (1.0 g/day for 29 days), resveratrol does not seem to have any negative side effects [33,34]. Otherwise, adverse symptoms such as nausea, vomiting, diarrhea and liver dysfunction in people with non-alcoholic fatty liver disease may develop at dosages of 2.5 g or higher per day [33,34]. It is interesting to note that lengthy clinical studies have revealed no significant adverse effects [35]. Indeed, studies have shown that resveratrol, whether taken as part of a multi-day dosing regimen or as a single dosage of up to 5 g/day, is safe and well-tolerated [36].

A study in France examined the impact of wine consumption (N = 184 participants) on short-term enjoyment. One group preferred wines with high alcohol levels, low acidity and fruity flavors, similar to those who enjoy sugary soft drinks. The study measured differences in taste preferences and purchasing habits between those who had tried the wines before and those who had not. The results showed that exposure to wine influenced consumer behavior and preferences, specifically in favor of fruity and alcoholic flavors. However, the study also found that single measurements cannot accurately predict future consumer choices [37]. Table 4 shows that a large number consume wine occasionally (1 glass): half of the group from FT, half of the group from MD and 25.71% from EECS. A relatively large number answered that they do not consume alcohol: 10 from MD and 17 from FT. But the respondents from EECS consume three glasses of wine occasionally in 14.29%, in comparison with the MD and FT groups where the percentage for this response is 0%.

Numerous experimental studies have studied the synergistic antioxidant action of caffeic acid with α-tocopherol and vitamin C. It was observed that under a diet rich in caffeic acid, lipoproteins were resistant to the action of antioxidants, and caffeic acid supplementation in the diet led to increased levels of α-tocopherol in plasma and lipoproteins [38]. Other studies have shown the ability of caffeic acid to reduce α-tocopherol [39,40] and the possibility of regeneration of caffeic acid from its radical by ascorbic acid [38]. The redox caffeic acid/ascorbate couple is the basis of many plant detoxification processes, demonstrating the possibility of a synergistic mechanism of action of α-tocopherol/caffeic acid/ascorbic acid [11].

Caffeic acid plays a crucial role in protecting cells from cell death, treating certain blood disorders and inhibiting the activity of a harmful enzyme found in snake venom [41]. It is also used in cosmetics and hair dyes as an antioxidant, antibacterial and anti-mutagenic agent. Therefore, it is important to be able to measure the amount of caffeic acid in various settings accurately. There are several sources of caffeic acid, including berries, oil and black olives, spices, coffee and certain vegetables [12].

The highest percentages for this question were for the “coffee” answer option, chosen by 90% of the respondents from Food and Tourism, 86% of the respondents from Medicine and 77.14% of the respondents from Electrical Engineering and Computer Science. In a smaller percentage, some chose that caffeic acid is also identifiable in berries and spices, according to Table 6.

Numerous clinical trials have been performed to highlight the effects of clinical acid [42,43]. Some of them report that it helps reduce inflammation, prevents the associated toxicity from chemotherapy and radiation treatments, prevents neurodegenerative diseases, including Parkinson’s disease, and can reduce the feeling of fatigue [44,45,46]. Analyzing Table 6, the study group responded in a large percentage that caffeic acid helps reduce fatigue: students from Medicine, 48%; those from Food and Tourism, 54% and those from Electrical Engineering and Computer Science, 42.86%. In total, 25.58% of EECS, 26% of FT students and 28% of MDs chose that caffeic acid helps reduce inflammation. Only in small percentages, 14% of those from FT, 25.71% of EECS and only 28% of those from MD chose that all variants are correct.

Numerous investigations revealed that large dosages of caffeic acid may have serious adverse consequences, such as carcinogenic effects [47] or an inhibition of embryo implantation [48].

Caffeic acid esters (50–150 mg), which have health benefits and nutritional effects, are found in coffee (200 mL) [49]. The quality of these effects depends on how the coffee is processed, packaged, handled after harvest and stored [50]. In Poland, approximately 82,000 tons of coffee were sold in 2017, prompting a study involving 800 participants from different regions of the country. This four-year research revealed that 85% of the participants preferred instant coffee, and 36% of them preferred to drink ground coffee once or twice a day [51]. Table 7 shows that the research participants from MD and FT prefer ground coffee at a percentage of 46–48%, but those from EECS prefer ground coffee in 28.57%. The quantity of consumed coffee is more than seven cups/week for a percentage of 22% from the MD group and 32% from the FT group, but a smaller percentage was registered in the EECS group with 8.57%. We can observe that a percentage of 38% from the MD group, 25.71% from EECS and 30% from FT do not consume coffee.

## 5. Conclusions

Following the administration of the questionnaire, the level of knowledge of the notions of pathology was better outlined among students in the Faculty of Medicine, but on the side of nutrition, students in the second study group (from the Faculty of Food and Tourism) showed a better level of knowledge developed. The notion of antioxidants is relatively well-known by both the MD and FT groups, but there is some missing information for the EECS group about their beneficial role. All groups managed to give examples of antioxidants and indicate most of their effects.

Coffee and wine are consumed by most of those selected, but young consumers do not know their antioxidant effects and benefits. Resveratrol and caffeic acid are antioxidant compounds less known among students in both the Faculty of Medicine and the Faculty of Food and Tourism. Following the administration of this questionnaire, it is recommended to have in-depth knowledge of these important antioxidants found in the daily diet, in terms of nutrition, and to consider different strategies to increase the level of knowledge. Special importance must be given to future similar studies of young consumers in other different cultural backgrounds, social statuses and lifestyle habits for multi- and inter-correlation studies.

## Figures and Tables

**Table 1:** Distribution of the answers of the study groups regarding their knowledge of antioxidants.

Item		MED		FT		EECS	
		No.	%	No.	%	No.	%
Antioxidants definition							
	Specific compounds that have the role of reducing the effects of free radicals	33	66.0	34	68.0	7	20
	Compounds that we can take from food	24	48.0	5	10.0	9	25.7
	Compounds that do not help the body to function within normal parameters	5	10.0	1	2.0	1	2.9
	Compounds that can stop the oxidation of other substances	20	40.0	20	40.0	25	71.4
	They give pathognomonic reactions once ingested	0	0.0	0	0.0	0	0
	I have never heard of this term	0	0.0	0	0.0	1	2.9
Example of antioxidants							
	Vitamin A	22	44.0	14	28.0	16	45.7
	Nicotine	1	2.0	0	0.0	3	8.6
	SOD	14	28.0	14	28.0	5	14.3
	Pollutants	0	0.0	0	0.0	1	2.9
	UV radiation	1	2.0	0	0.0	2	5.7
	Vitamin C	34	68.0	33	66.0	20	57.1
	Aluminum	1	2.0	1	2.0	1	2.9
	Glutathione peroxidase	6	12.0	2	4.0	5	14.3
	Caffeic acid	24	48.0	25	50.0	21	60.0
	Resveratrol	26	52.0	22	44.0	14	40.0
	Coenzyme Q	18	36.0	19	38.0	8	22.9
	Vitamin E	19	38.0	20	40.0	16	45.7
Examples of antioxidants sources							
	Carrot	43	86.0	41	82.0	27	77.1
	Refined sugar	1	2.0	0	0.0	1	2.9
	Beans	19	38.0	18	36.0	18	51.4
	Carbonated drinks	2	4.0	3	6.0	11	31.4
	Hydrogenated oils	5	10.0	5	10.0	4	11.4
	Chocolate	18	36.0	12	24.0	10	28.6
	Eggs	11	22.0	10	20.0	15	42.9
Favorite forms for taking antioxidants							
	Food supplements	4	8.0	3	6.0	10	28.6
	Vegetables	41	82.0	40	80.0	26	74.3
	Fruits	41	82.0	40	80.0	27	77.1
	Teas	20	40.0	12	24.0	4	11.4
	Others	1	2.0	1	2.0	1	2.9
The beneficial effects of antioxidants							
	Prevention of cardiovascular diseases	14	28.0	12	24.0	10	28.6
	Prevent aging	22	44.0	17	34.0	6	17.1
	Chemoprevention for cancer	10	20.0	5	10.0	3	8.6
	It doesn’t help	0	0.0	0	0.0	20	57.1
	No answer	0	0.0	2	4.0	0	0

**Table 2:** The assessment of young consumers concerning healthy nutrition habits.

Item	Group	% of the Positive Answer Strong Disagreement—Neither against nor Agree—Strong Agreement						
		1	2	3	4	5	6	7
I’m interested in how healthy is what I eat	MD	0.0	4.0	2.0	10.0	18.0	16.0	50.0
	FT	2.0	4.0	0.0	14.0	14.0	16.0	50.0
	EECS	2.9	0.0	5.7	14.3	25.7	25.7	25.7
I always follow a healthy and balanced diet	MD	0.0	8.0	16.0	34.0	26. 0	8.0	8.0
	FT	8.0	20.0	14.0	30.0	10.0	14.0	4.0
	EECS	5.7	8.6	20.0	37.1	8.6	8.6	11.4
It is important for me that the diet is low in fat	MD	4.0	8.0	20.0	20.0	12.0	16.0	20.0
	FT	10.0	8.0	10.0	38.0	16.0	2.0	16.0
	EECS	5.7	17.1	17.1	28.6	14.3	8.6	8.6
It is important for me that the daily diet contains many vitamins and minerals	MD	0.0	0.0	10.0	18.0	30.0	12.0	30.0
	FT	0.0	0.0	12.0	26.0	26.0	14.0	22.0
	EECS	0.0	8.6	5.7	31.4	20.0	20.0	14.3
I eat what I like and I don’t really care how healthy it is what I eat	MD	22.0	4.0	14.0	28.0	10.0	16.0	6.0
	FT	16.0	18.0	2.0	30.0	14.0	4.0	16.0
	EECS	8.6	20.0	5.7	22.9	20.0	17.1	5.7
I do not avoid certain foods, even if they can affect my health	MD	24.0	8.0	12.0	24.0	22.0	6.0	4.0
	FT	24.0	8.0	16.0	16.0	10.0	14.0	12.0
	EECS	8.6	20.0	5.7	22.9	20.0	17.1	5.7
How healthy is what I eat—has little impact on food choices	MD	30.0	14.0	14.0	24.0	10.0	4.0	4.0
	FT	12.0	18.0	10.0	22.0	8.0	12.0	18.0
	EECS	5.7	8.6	22.9	22.9	17.1	8.6	14.3
It doesn’t matter to me how unhealthy the snacks are	MD	40.0	12.0	22.0	14.0	10.0	2.0	0.0
	FT	26.0	14.0	14.0	22.0	4.0	8.0	12.0
	EECS	22.9	14.3	11.4	22.9	5.7	11.4	11.4

**Table 3:** Distribution of study group responses on the knowledge of resveratrol and its benefits.

Item		MD		FT		EECS	
		No.	%	No.	%	No.	%
Source of knowledge of the resveratrol effects							
	No	36	72.0	26	52.0	32	91.4
	Yes, from the doctor	1	2.0	1	2.0	0	0.0
	Yes, from the internet	5	10.0	7	14.0	2	5.7
	Yes, from the TV	1	2.0	1	2.0	1	2.9
	Yes, from pharmacies	4	8.0	1	2.0	0	0.0
	Yes, from the teachers	5	10.0	17	34.0	0	0.0
Possible sources of resveratrol							
	Grapes	35	70.0	34	68.0	15	42.9
	Peanuts	4	8.0	5	10.0	14	40.0
	Cranberries	15	30.0	10	20.0	16	45.7
	Blueberries	24	48.0	11	22.0	9	25.7
	Lemon	6	12.0	2	4.0	5	14.3
	Spices	4	8.0	5	10.0	2	5.7
	Mulberries	11	22.0	6	12.0	9	25.7
	No answer	0	0.0	7	14.0	0	0.0
Benefits of resveratrol							
	Anti-inflammatory	23	46.0	25	50.0	15	42.9
	Cardioprotective	20	40.0	17	34.0	12	34.3
	Reduces obesity	17	34.0	4	8.0	10	28.6
	Neuroprotective	13	26.0	4	8.0	8	22.9
	Prevents aging	27	54.0	19	38.0	4	11.4
	Improves vision	7	14.0	1	2.0	4	11.4
	Increases the risk of Alzheimer’s	0	0.0	1	2.0	0	0.0
	They did not answer	0	0.0	7	14.0	0	0.0
What are the therapeutic benefits of resveratrol and its derivatives?							
	Chemotherapeutic in breast and lung cancer	15	30.0	7	14.0	8	22.9
	Inhibits the growth of malignant tumors, such as breast, colon, leukemia, myeloma	25	50.0	20	40.0	16	45.7
	Enhances cell proliferation	10	20.0	3	6.0	8	22.9
	Anti-HIV1	2	4.0	3	6.0	1	2.9
	Antibacterial	19	38.0	13	26.0	8	22.9
	Triggers cytochrome activity P-450	13	26.0	0	0.0	3	8.6
	Accentuates the process of atherosclerosis and hypertension	3	6.0	6	12.0	4	11.4
	No answer	0	0.0	9	18.0	0	0.0

**Table 4:** Distribution of the answers of the study groups regarding the consumption frequency of alcoholic drinks (1 glass = 250 mL).

Consumption Frequency of Alcoholic Drinks		I Don’t Drink Alcohol (%)			1 Glass (%)			2 Glasses (%)			3 Glasses (%)		
		MD	FT	EECS	MD	FT	EECS	MD	FT	EECS	MD	FT	EECS
	Daily				6.0	2.0	0.0	2.0	0.0	2.9	0.0	0.0	2.9
	Weekly				0.0	2.0	0.0	12.0	6.0	2.9	0.0	0.0	8.6
	Occasionally				50.0	50.0	25.7	8.0	2.0	20.0	2.0	2.0	14.3
	I don’t drink alcohol	20.0	34.0	17.1									

**Table 5:** Distribution of the answers of the study groups regarding the criteria of choice and consumption of wine.

Item		MD		FT		EECS	
		No.	%	No.	%	No.	%
Favorite type of wine							
	I prefer commercialized white wine	6	12.0	9	18.0	2	5.7
	I prefer commercialized red wine	13	26.0	11	22.0	10	28.6
	I prefer eco-white wine	4	8.0	8	16.0	10	28.6
	I prefer eco-red wine	21	42.0	17	34.0	13	37.1
	I don’t drink wine	10	20.0	14	28.0	5	14.3
	They didn’t answer	0	0.0	1	2.0	0	0.0
Wine consumption criteria							
	Affordable price	6	12.0	3	6.0	11	31.4
	Brand	12	24.0	8	16.0	9	25.7
	Attractive packaging	2	4.0	0	0.0	2	5.7
	Category (dry, semi-dry, sweet)	37	74.0	22	44.0	21	60.0
	Wine variety (merlot, etc.)	16	32.0	18	36.0	13	37.1
	Shape of the bottle	1	2.0	0	0.0	0	0.0
	The area from which the grapes come	10	20.0	13	26.0	2	5.7
	No answer	0	0.0	13	26.0	1	2.9
Reasons for wine consumption							
	It’s an effective energizer	8	16.0	3	6.0	5	14.3
	It is an effective antidepressant	8	16.0	7	14.0	6	17.1
	Promoting mode	2	4.0	2	4.0	0	0.0
	Friends influence	14	28.0	13	26.0	12	34.3
	Price reductions	3	6.0	0	0.0	1	2.9
	Free-of-charge product	1	2.0	1	2.0	0	0.0
	I don’t drink wine	11	22.0	15	30.0	4	11.4
	Others	8	16.0	10	20.0	11	31.4
	No answer	0	0.0	2	4.0	0	0.0
Average amount allocated monthly for the purchase/consumption of wine *							
	30–50 RON	28	56.0	20	40.0	22	62.9
	50–150 RON	11	22.0	15	30.0	6	17.1
	Over 150 RON	3	6.0	1	2.0	4	11.4
	No answer	8	16.0	14	28.0	2	5.7

1 RON = 0.20 euro (February 2024 *).

**Table 6:** Distribution of study group responses on the knowledge of caffeic acid and its benefits.

Item		MD		FT		EECS	
		No.	%	No.	%	No.	%
Caffeic acid sources							
	Berries	11	22.0	10	20.0	5	14.3
	Oil, black olives	4	8.0	6	12.0	3	8.6
	Spices	12	24.0	8	16.0	5	14.3
	Coffee	43	86.0	45	90.0	27	77.1
	Vegetables	7	14.0	3	6.0	5	14.3
	Oranges	2	4.0	2	4.0	2	5.7
	Corn flour	1	2.0	0	0.0	0	0.0
	Cheese	0	0.0	0	0.0	0	0.0
Benefits of caffeic acid							
	Reduces inflammation	14	28.0	13	26.0	10	28.6
	Prevention of toxicity associated with chemotherapy and radiation	5	10.0	2	4.0	4	11.4
	Prevents neurodegenerative diseases, such as Parkinson’s disease	11	22.0	5	10.0	5	14.3
	Reduces fatigue	24	48.0	27	54.0	15	42.9
	All options are correct	14	28.0	7	14.0	9	25.7
	I don’t know any benefit of caffeic acid	0	0.0	1	2.0	3	8.6
	They didn’t answer	0	0.0	1	2.0	0	0.0
The preferred form of caffeic acid consumption							
	In the form of pharmaceutical supplements	0	0.0	2	4.0	0	0.0
	Coffee	28	56.0	27	54.0	22	62.9
	Diversified fruits containing a considerable amount of caffeic acid	9	18.0	6	12.0	6	17.1
	I’m not interested	16	32.0	20	40.0	8	22.9

**Table 7:** Distribution of the answers of the study groups regarding the criteria of choice and consumption of coffee.

		MD		FT		EECS	
		No.	%	No.	%	No.	%
The amount of coffee consumed weekly							
	More than 7 cups/week	11	22.0	16	32.0	3	8.6
	7 cups/week	8	16.0	4	8.0	6	17.1
	Less than 5 cups/week	10	20.0	7	14.0	11	31.4
	I didn’t noticed	1	2.0	3	6.0	1	2.9
	I don’t drink coffee	19	38.0	15	30.0	9	25.7
	Another answer	1	2.0	5	10.0	6	17.1
Reason for coffee consumption							
	I like the taste	15	30.0	18	36.0	19	54.3
	I consume coffee when I feel stressed, fatigued	16	32.0	17	34.0	12	34.3
	It has become a habit	11	22.0	9	18.0	4	11.4
	I like the odor	8	16.0	12	24.0	6	17.1
	I don’t drink coffee	19	38.0	16	32.0	7	20.0
The form in which coffee is consumed							
	Coffee beans	3	6.0	5	10.0	8	22.9
	Ground coffee	24	48.0	23	46.0	10	28.6
	Instant coffee	7	14.0	10	20.0	9	25.7
	Coffee capsules	4	8.0	2	4.0	7	20.0
	Another form	0	0.0	0	0.0	2	5.7
	I don’t drink coffee	19	38.0	16	32.0	8	22.9
Criteria for choosing coffee for consumption							
	Price	2	4.0	2	4.0	6	17.1
	Brand	10	20.0	12	24.0	7	20.0
	Aroma, concentration	22	44.0	20	40.0	18	51.4
	The color of the packaging	0	0.0	0	0.0	0	0.0
	Package weight (50 g, 100 g, 250 g, 500 g, 1 kg)	1	2.0	3	6.0	1	2.9
	Friends/family recommendation	4	8.0	3	6.0	6	17.1
	I don’t prefer a certain coffee, I drink any kind of coffee	4	8.0	5	10.0	5	14.3
	I don’t drink coffee	19	38.0	17	34.0	7	20.0
Coffee selection criteria							
	Must be eco	2	4.0	5	10.0	5	14.3
	To have an affordable price	8	16.0	10	20.0	9	25.7
	Attractive packaging	1	2.0	2	4.0	1	2.9
	Package weight must be high	0	0.0	1	2.0	1	2.9
	Must be very fragrant	23	46.0	23	46.0	16	45.7
	Must be less fragrant	2	4.0	0	0.0	1	2.9
	Must be from a specific manufacturing company	6	12.0	4	8.0	3	8.6
	I don’t drink coffee	19	38.0	13	26.0	7	20.0
	They didn’t answer	0	0.0	2	4.0	0	0.0
Average amount allocated for coffee consumption/week *							
	10–30 RON	32	64.0	27	54.0	21	60.0
	30–70 RON	6	12.0	10	20.0	6	17.1
	Over 70 RON	2	4.0	1	2.0	6	17.1
	No answer	10	20.0	12	24.0	2	5.7

1 RON = 0.20 euro (February 2024 *).

## Data Availability

The original contributions presented in the study are included in the article, further inquiries can be directed to the corresponding author.

## References

[1] Paul B., Masih I., Deopujari J., Charpentier C. (1999). Occurrence of resveratrol and pterostilbene in age-old darakchasava, an ayurvedic medicine from India. J. Ethnopharmacol..

[2] Langcake P.W.V.M., McCarthy W.V. (1979). The relationship of resveratrol production to infection of grapevine leaves by Botrytis cinerea. Vitis.

[3] Trela B.C., Waterhouse A.L. (1996). Resveratrol: Isomeric molar absorptivities and stability. J. Agric. Food Chem..

[4] Jasiński M., Jasińska L., Ogrodowczyk M. (2013). Resveratrol in prostate diseases—A short review. Cent. Eur. J. Urol..

[5] Bellaver B., Souza D.G., Souza D.O., Quincozes-Santos A. (2014). Resveratrol increases antioxidant defenses and decreases proinflammatory cytokines in hippocampal astrocyte cultures from newborn, adult and aged Wistar rats. Toxicol. Vitr..

[6] Vergara D., De Domenico S., Tinelli A., Stanca E., Del Mercato L.L., Giudetti A.M., Simeone P., Guazzelli N., Lessi M., Manzini C. (2017). Anticancer effects of novel resveratrol analogues on human ovarian cancer cells. Mol. Biosyst..

[7] Sinha D., Sarkar N., Biswas J., Bishayee A. (2016). Resveratrol for breast cancer prevention and therapy: Preclinical evidence and molecular mechanisms. Semin. Cancer Biol..

[8] Wang W., Zhang L., Chen T., Guo W., Bao X., Wang D., Ren B., Wang H., Li Y., Wang Y. (2017). Anticancer effects of resveratrol-loaded solid lipid nanoparticles on human breast cancer cells. Molecules.

[9] Stafford H.A. (1974). The metabolism of aromatic compounds. Ann. Rev. Plant Physiol..

[10] Rice-Evans C.A., Miller N.J., Paganga G. (1996). Structure-antioxidant activity relationships of flavonoids and phenolic acids. Free Radic. Biol. Med..

[11] Samoilă O.C. (2008). Posibilități de reglare a nivelului speciilor reactive ale oxigenului în organismul uman: Aplicații în ingineria alimentară și medicină. Ph.D. Thesis.

[12] Fan D., Jia L., Xiang H., Peng M., Li H., Shi S. (2017). Synthesis and characterization of hollow porous molecular imprinted polymers for the selective extraction and determination of caffeic acid in fruit samples. Food Chem..

[13] Hapiot P., Neudeck A., Pinson J., Fulcrand H., Neta P., Rolando C. (1996). Oxidation of caffeic acid and related hydroxycinnamic acids. J. Electroanal. Chem..

[14] Akomolafe S.F., Akinyemi A.J., Ogunsuyi O.B., Oyeleye S.I., Oboh G., Adeoyo O.O., Allismith Y.R. (2017). Effect of caffeine, caffeic acid and their various combinations on enzymes of cholinergic, monoaminergic and purinergic systems critical to neurodegeneration in rat brain—In vitro. Neurotoxicology.

[15] Khan A.Q., Khan R., Qamar W., Lateef A., Ali F., Tahir M., Sultana S. (2012). Caffeic acid attenuates 12-O-tetradecanoyl-phorbol-13-acetate (TPA)-induced NF-κB and COX-2 expression in mouse skin: Abrogation of oxidative stress, inflammatory responses and proinflammatory cytokine production. Food Chem. Toxicol..

[16] Weng C.J., Yen G.C. (2012). Chemopreventive effects of dietary phytochemicals against cancer invasion and metastasis: Phenolic acids, monophenol, polyphenol, and their derivatives. Cancer Treat. Rev..

[17] Oh J., Jo H., Cho A.R., Kim S.J., Han J. (2013). Antioxidant and antimicrobial activities of various leafy herbal teas. Food Control.

[18] Wang F., Yang J. (2012). A comparative study of caffeic acid and a novel caffeic acid conjugate SMND-309 on antioxidant properties in vitro. LWT—Food Sci. Technol..

[19] Choudhury R., Srai S.K., Debnam E., Rice-Evans C.A. (1999). Urinary excretion of hydroxycinnamates and flavonoids after oral and intravenous administration. Free Radic. Biol. Med..

[20] Bourne L., Rice-Evans C.A. (1998). Urinary detection of hydroxycinnamates and flavonoids in human after high dietary intake of fruit. Free Radic. Res..

[21] El-Seedi H.R., El-Said A.M., Khalifa S.A., Göransson U., Bohlin L., Borg-Karlson A.K., Verpoorte R. (2012). Biosynthesis, natural sources, dietary intake, pharmacokinetic properties, and biological activities of hydroxycinnamic acids. J. Agric. Food Chem..

[22] Pavlíková N. (2022). Caffeic acid and diseases-mechanisms of action. Int. J. Mol. Sci..

[23] Scalbert A., Johnson I.T., Saltmarsh M. (2005). Polyphenols: Antioxidants and beyond. Am. J. Clin. Nutr..

[24] Luzardo O.P., Badea M., Zumbado M., Rogozea L., Floroian L., Ilea A., Moga M., Sechel G., Boada L.D., Henríquez-Hernández L.A. (2019). Body burden of organohalogenated pollutants and polycyclic aromatic hydrocarbons in Romanian population: Influence of age, gender, body mass index, and habitat. Sci. Total Environ..

[25] Murias M., Jäger W., Handler N., Erker T., Horvath Z., Szekeres T., Nohl H., Gille L. (2005). Antioxidant, prooxidant and cytotoxic activity of hydroxylated resveratrol analogues: Structure-activity relationship. Biochem. Pharmacol..

[26] Sanchez R., Gonzales M.R., Fernandez-Fernandez E., Rodriguez-Nogales J.M., Martin P. (2020). Relationships between chlorophyll content of vine leaves, predawn leaf water potential at veraison, and chemical and sensory attributes of wine. J. Sci. Food Agric..

[27] Tonacci A., Taglieri I., Sanmartin C., Bileci L., Crifaci G., Ferroni G., Braceschi G.P., Odello L., Venturi F. (2023). Taste the emotions: Pilot for a novel, sensors-based approach to emotional analysis during coffee tasting. J. Sci. Food Agric..

[28] Ndhlala A.R., Moyo M., Van Staden J. (2010). Natural antioxidants: Fascinating or mythical biomolecules?. Molecules.

[29] Zulueta A., Esteve M.J., Frígola A. (2009). ORAC and TEAC assays comparison to measure the antioxidant capacity of food products. Food Chem..

[30] Aggarwal B.B., Shishodia S. (2006). Resveratrol in Health and Disease.

[31] Xu D.P., Li Y., Meng X., Zhou T., Zhou Y., Zheng J., Zhang J.J., Li H.B. (2017). Natural antioxidants in foods and medicinal plants: Extraction, assessment and resources. Int. J. Mol. Sci..

[32] Pastor R.F., Restani P., Di Lorenzo C., Orgiu F., Teissedre P.L., Stockley C., Ruf J.C., Quini C.I., Garcìa Tejedor N., Gargantini R. (2019). Resveratrol, human health and winemaking perspectives. Crit. Rev. Food Sci. Nutr..

[33] Salehi B., Mishra A.P., Nigam M., Sener B., Kilic M., Sharifi-Rad M., Fokou P.V.T., Martins N., Sharifi-Rad J. (2018). Resveratrol: A double-edged sword in health benefits. Biomedicines.

[34] Brown V.A., Patel K.R., Viskaduraki M., Crowell J.A., Perloff M., Booth T.D., Vasilinin G., Sen A., Schinas A.M., Piccirilli G. (2010). Repeat dose study of the cancer chemopreventive agent resveratrol in healthy volunteers: Safety, pharmacokinetics, and effect on the insulin-like growth factor axis. Cancer Res..

[35] Tomé-Carneiro J., Gonzálvez M., Larrosa M., Yáñez-Gascón M.J., García-Almagro F.J., Ruiz-Ros J.A., Tomás-Barberán F.A., García-Conesa M.T., Espín J.C. (2013). Grape resveratrol increases serum adiponectin and downregulates inflammatory genes in peripheral blood mononuclear cells: A triple-blind, placebo-controlled, one-year clinical trial in patients with stable coronary artery disease. Cardiovasc. Drugs Ther..

[36] Patel K.R., Scott E., Brown V.A., Gescher A.J., Steward W.P., Brown K. (2011). Clinical trials of resveratrol. Ann. N. Y. Acad. Sci..

[37] Cichewicz R.H., Kouzi S.A. (2002). Resveratrol oligomers: Structure, chemistry, and biological activity. Stud. Nat. Prod. Chem..

[38] Ma B.N., Li X.J. (2020). Resveratrol extracted from Chinese herbal medicines: A novel therapeutic strategy for lung diseases. Chin. Herb. Med..

[39] Tempere S., Pérès S., Espinoza A.F., Darriet P., Giraud-Héraud E., Pons A. (2019). Consumer preferences for different red wine styles and repeated exposure effects. Food Qual. Prefer..

[40] Stocker R., Bowry V.W. (1996). Tocopherol-Mediated Peroxidation of Lipoprotein Lipids and Its Inhibition by Co-Antioxidants.

[41] Shimabuku P.S., Fernandes C.A.H., Magro A.J., Costa T.R., Soares A.M., Fontes M.R.M. (2011). Crystallization and preliminary X-ray diffraction analysis of a Lys49-phospholipase A2 complexed with caffeic acid, a molecule with inhibitory properties against snake venoms. Acta Crystallogr. Sect. F Struct. Biol. Cryst. Commun..

[42] Witting P.K., Westerlund C., Stockerl R. (1996). A rapid and simple screening test for potential inhibitors of tocopherol-mediated peroxidation of LDL lipids. J. Lipid Res..

[43] Laranjinha J., Cadenas E. (1999). Redox cycles of caffeic acid, α-tocopherol, and ascorbate: Implications for protection of low-density lipoproteins against oxidation. IUBMB Life.

[44] Wadhwa R., Nigam N., Bhargava P., Dhanjal J.K., Goyal S., Grover A., Sundar D., Ishida Y., Terao K., Kaul S.C. (2016). Molecular characterization and enhancement of anticancer activity of caffeic acid phenethyl ester by γ cyclodextrin. J. Cancer.

[45] Grigorescu S., Cazan A.M., Rogozea L., Grigorescu D.O. (2022). Predictive Factors of the Burnout Syndrome Occurrence in the Healthcare Workers during the COVID-19 Pandemic. Front. Med..

[46] Mateescu M.C., Grigorescu S., Socea B., Bloanca V., Grigorescu O.D. (2023). Contribution to the personalized management of the nosocomial infections: A new Paradigm regarding the influence of the community microbial environment on the incidence of the healthcare-associated infections (HAI) in emergency hospital surgical departments. J. Pers. Med..

[47] Hagiwara A., Takahashi S., Ogawa K., Shirai T., Ito N. (1991). Forestomach and kidney carcinogenicity of caffeic acid in F344 rats and C57BL/6N × C3H/HeN F_1_ mice. Cancer Res..

[48] Liu Y., Qiu S., Wang L., Zhang N., Shi Y., Zhou H., Liu X., Shao L., Liu X., Chen J. (2019). Reproductive and developmental toxicity study of caffeic acid in mice. Food Chem. Toxicol..

[49] Scalbert A., Williamson G. (2000). Dietary Intakeand Bioavailability of Polyphenols. J. Nutr..

[50] Pérez-Gregorio M.R., García-Falcón M.S., Simal-Gándara J. (2011). Flavonoids changes in fresh-cut onions during storage in different packaging systems. Food Chem..

[51] Wrablewski Å., Maciejewski G., Mokrysz S. Consumers on the coffee market in central European countries: The case of Poland. Proceedings of the 33rd International Business Information Management Association Conference IBIMA 2019 Education Excellence and Innovation Management through Vision 2020.

